# Changes in the Nasal Microbiota of Pigs Following Single or Co-Infection with Porcine Reproductive and Respiratory Syndrome and Swine Influenza A Viruses

**DOI:** 10.3390/pathogens10101225

**Published:** 2021-09-22

**Authors:** Tiphany Chrun, Joy Leng, Roberto M. La Ragione, Simon P. Graham, Elma Tchilian

**Affiliations:** 1 The Pirbright Institute, Woking GU24 0NF, UK; simon.graham@pirbright.ac.uk (S.P.G.); elma.tchilian@pirbright.ac.uk (E.T.); 2 School of Veterinary Medicine, University of Surrey, Guildford GU2 7AL, UK; r.laragione@surrey.ac.uk

**Keywords:** swine influenza A virus, porcine reproductive and respiratory syndrome virus, nasal microbiota, co-infection, next-generation sequencing 16S rRNA

## Abstract

Host-microbiota interactions are important in shaping immune responses that have the potential to influence the outcome of pathogen infection. However, most studies have focused on the gut microbiota and its possible association with disease outcome, while the role of the nasal microbiota and respiratory pathogen infection has been less well studied. Here we examined changes in the composition of the nasal microbiota of pigs following experimental infection with porcine reproductive and respiratory syndrome virus 2 (PRRSV-2), swine influenza A H3N2 virus (H3N2) or both viruses. DNA extracted from nasal swabs were subjected to 16S rRNA sequencing to study the composition of the nasal microbiota. Bacterial richness fluctuated in all groups, with a slight reduction in pigs singly infected with PRRSV-2 and H3N2 during the first 5 days of infection compared to uninfected controls. In contrast, nasal bacterial richness remained relatively stable after PRRSV-2/H3N2 co-infection. PRRSV-2 and H3N2, alone or in combination differentially altered the abundance and distribution of bacterial families. Single and co-infection with PRRSV-2 or H3N2 was associated with the expansion of the *Neisseriaceae* family. A positive correlation between H3N2 viral load and the relative abundance of the *Neisseriaceae* was observed. However, further mechanistic studies are required to understand the significance of the changes in specific bacterial families following these viral infections.

## 1. Introduction

In the last 10 years extensive studies undertaken in humans and farm animals have indicated the important role that microbiota diversity and composition plays in the control of infectious diseases. Numerous studies have shown the importance of the gut microbiota on immunity to respiratory infections, although there is little knowledge as to how these two complex systems interact [1]. Indeed, antibiotic induced dysbiosis of the intestinal microbiota in mice resulted in impaired immune responses, leading to increased influenza A virus (IAV) [2,3] or *Mycobacterium tuberculosis* [4] burdens in the lungs after challenge. Studies in germ-free or antibiotic treated animals have further demonstrated that endogenous gastrointestinal microbiota are beneficial for combatting pulmonary infections. For example, fecal microbiota from healthy adult boar administered orally to nursery pigs prior to challenge with *Mycoplasma hyopneumoniae* resulted in a rapid antibody response, decreased gross lesions and less coughing [5]. Oral administration of lactic acid bacteria *Enterococcus faecalis* in pigs led to increased IFN-γ and TNF expression in secondary lymphoid organs [6]. In contrast to the gut microbiota, there has been less evaluation of the role of the respiratory tract microbiota in the outcome of viral infection in pigs. The oropharynx of pigs affected with porcine respiratory disease complex (PRDC), caused by a combination of viral and/or bacterial pathogens and environmental stressors [7], displayed higher abundance of *Moraxella*, *Veillonella*, and *Porphyromonas* genera compared to healthy pigs [8]. Another study showed that the lungs from PRDC-infected pigs were enriched for *Streptococcus*, *Haemophilus*, *Pasteurella*, and *Bordetella* genera [9]. To our knowledge, no studies have thus far investigated the nasal microbiota communities in pigs after infection with respiratory viruses.

Sequencing of the bacterial 16S rRNA genes allows for the study of complex bacterial communities such as those found within the gut or respiratory tract without the need for culturing and identifies fastidious bacterial groups that cannot be cultured within a laboratory setting. This approach was previously employed to understand how the nasal microbiota develops in the early life of pigs, with the bacterial populations stabilizing around three weeks of age [10]. It has also been used to elucidate changes in the porcine respiratory microbiota linked with methicillin resistant *Staphylococcus aureus* (MRSA) carriage [11], food supplementation used to control the occurrence of *Streptococcus suis* associated disease [12] and on farms that had recurring *Mycoplasma hyorhinis* infections [13].

Multiple infections of pigs are more frequent than single infections in the field [14,15]. Among the PRDC associated pathogens, porcine reproductive and respiratory syndrome virus (PRRSV) and swine influenza A virus (swIAV) are major contributors, reaching up to 35.4% and 22.2%, respectively, of aetiologic agents responsible of respiratory diseases sampled in the field [15]. PRRSV is an enveloped, single stranded, positive sense RNA virus belonging to the *Arteriviridae* family [16] and exists as two species, PRRSV-1 (*Betaarterivirus suid 1*) and PRRSV-2 (*Betaarterivirus suid 2*), which are responsible for the porcine reproductive and respiratory syndrome (PRRS) in pigs [17]. This disease has considerable economic impact on the global pig industry, with annual economic loss estimated at US$664 million in the USA alone [18]. PRRS is characterized by reproductive failure in sows, respiratory disorders in young pigs and impairment of growth performance [19]. Influenza A virus is an enveloped single stranded segmented negative RNA virus within the *Orthomyxoviridae* family [16] and is a global threat for humans and livestock species. H1N1, H1N2, and H3N2 subtypes of swIAV are commonly circulating in pigs [20]. As in humans, swine influenza can be subclinical or can cause acute respiratory disorders associated with secondary bacterial infections, fever, and loss of appetite, which affects the productivity of growing pigs [21].

In this study, we examined the changes between the nasal commensal microbiota and porcine respiratory viruses which may be encountered in the upper respiratory tract. The study took advantage of nasal swabs taken as part of an experimental co-infection study with PRRSV-2 and swIAV H3N2 (H3N2) in pigs [22]. Next-generation sequencing of the bacterial 16S rRNA gene was performed to identify and quantify microbiota changes in nasal swabs of uninfected controls, single PRRSV-2 or H3N2 infected or PRRSV-2/H3N2 co-infected groups.

## 2. Results

### 2.1. Effects of Single and Viral Co-Infection on Nasal Microbiota Richness

**Figure 1:**
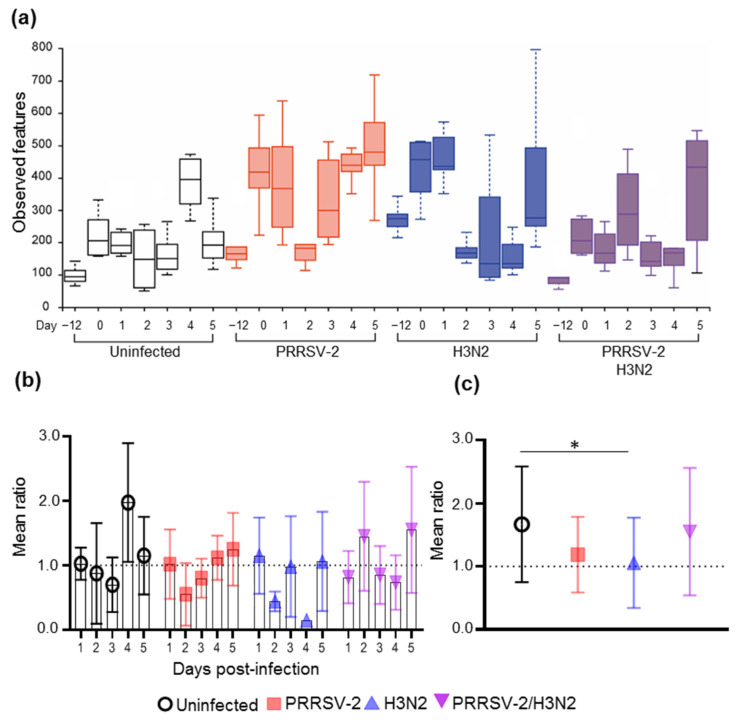
Nasal microbiota richness before and after single and co-infection with PRRSV-2 and H3N2. Nasal swabs were collected before the viral challenge (day −12 and 0) and for 5 consecutive days post-challenge from naïve uninfected, PRRSV-2, H3N2 and PRRSV-2/H3N2 co-infected pigs (*n* = 6/group). (**a**) Bacterial richness boxplots of each group over time; (**b**) Ratio of bacterial richness after challenge normalized to day 0 (dashed line); (**c**) Ratio of bacterial richness from day 1 to day 5 for each group. Each symbol represents the mean ratio of a group and bars indicate ± SD (*n* = 4–6 per group). Comparisons were made using Kruskal-Wallis test and asterisks indicate significant differences (* *p* < 0.05).

To evaluate how the nasal microbiota was affected after experimental infection of pigs with PRRSV-2, H3N2, or simultaneously with both viruses PRRSV-2/H3N2, nasal swab samples collected −12, 0, 1, 2, 3, 4 and 5 days post-infection (dpi) were analyzed by next-generation sequencing of the V4–V5 region of 16S rRNA. Controls were samples from naïve untreated (uninfected) animals collected at the same timepoints throughout the study. Bacterial richness, measured as the number of observed operational taxonomic units (OTUs) by features, was calculated for all samples to assess the differences in bacterial richness. The number of observed OTUs for each of the samples pre/post-infection are indicated in Figure 1. Each individual pig displayed a different number of OTUs. On average 12 days before the challenge (−12 dpi), the uninfected, PRRSV-2, H3N2 and PRRSV-2/H3N2 co-infected groups presented with a mean of 96, 185, 265 and 86 OTUs, respectively increasing to 217, 402, 450 and 252 OTUs, respectively at day 0 (Figure 1a). Regardless of the treatment, the bacterial richness within each group changed over time. To better capture whether the viral infection altered the microbiota richness, the ratio of OTUs at 1, 2, 3, 4 and 5 dpi was calculated after normalization of the number of OTUs at 0 dpi for each pig (Figure 1b,c). A greater reduction of the bacterial richness was observed in the singly infected groups, particularly in the H3N2 infected group at day 2 and 4 dpi (0.4 and 0.2 respectively) (Figure 1b,c).

### 2.2. Description of Nasal Microbiota Communities in Healthy Pigs

**Figure 2:**
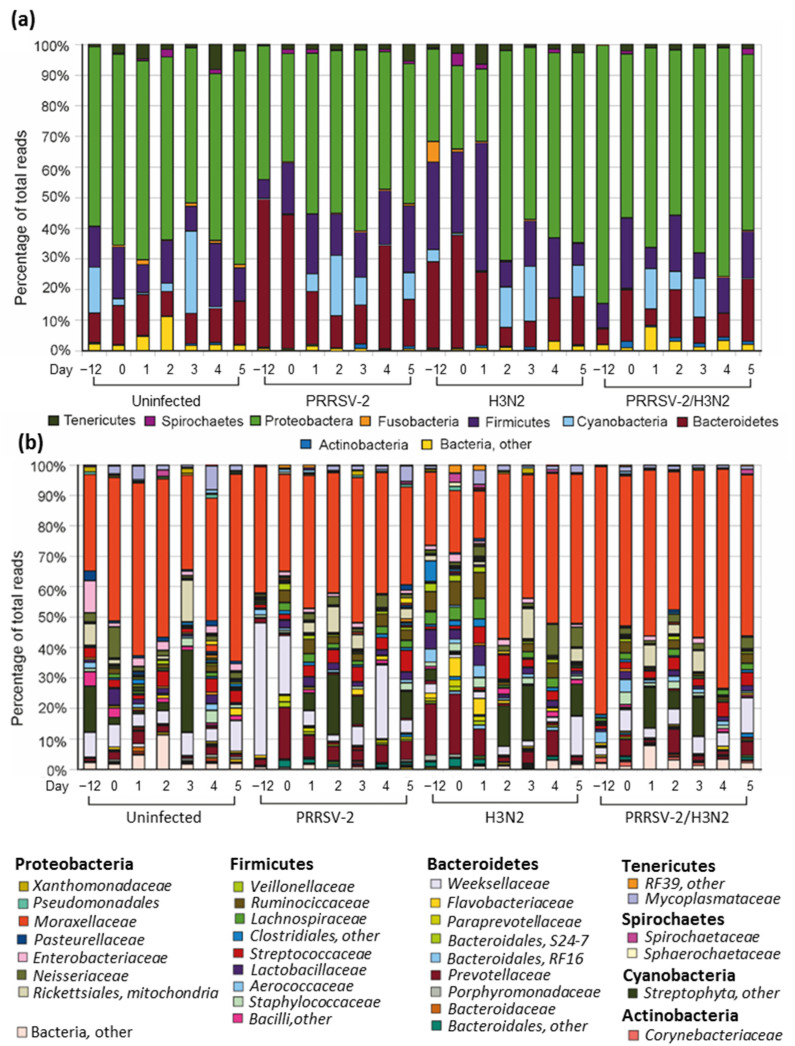
Composition of nasal microbiota before and after single or co-infection with PRRSV-2 and H3N2. The mean relative abundance (%) of different OTUs at phylum (**a**) and family; (**b**) levels.

To analyze the main phyla comprising the microbiota of the nasal cavity in healthy pigs, the average relative abundance was calculated in samples taken before the challenge (0 dpi) in all groups (Figure 2a, Supplementary Tables S1 and S2).

Despite a proportion of unassigned sequences (Bacteria, other 23.9%), the porcine nasal microbiota was mainly composed of 5 phyla: Protobacteria (48.3%), Bacteroidetes (21.7%), Firmicutes (16.2%), Cyanobacteria (5.2%) and Ternicutes (1.5%) (Supplementary Table S1). At the family level, the mean relative abundance in each day in all groups are shown in Figure 2b. In all pigs prior the challenge on average, *Moraxellaceae* (38.9%) was the most abundant followed by *Prevotellaceae* (9.2%), *Weeksellaceae* (7.3%), *Streptophyla* (4.5%), *Ruminococcaceae* (3.6%), *Lactobacillaceae* (3.3%), *Neisseriaceae* (2.4%), *Aerococcaceae* (2.4%), *Enterobacteriaceae* (2.3%), *Rickettsiales, mitochondria* (2.2%), *Lachnospiraceae* (2.2%), *Streptococcaceae* (2.0%), *Staphylococcaceae* (1.6%), *Veillonellaceae* (1.4%)*, Paraprevotellaceae* (1.0%) and *Mycoplasmataceae* (0.8%) (Supplementary Table S2). Other bacterial families that contain opportunistic bacterial pathogens commonly associated with respiratory diseases in pigs, such as, *Pseudomonadaceae* and *Mycoplasmataceae* represent <1% of the nasal microbiota [7,23]. These data indicated that bacterial communities fluctuated prior to the experimental viral challenge with Proteobacteria and *Moraxellaceae* comprising 25% or more of the identifiable bacterial reads (Figure 2a and Supplementary Table S1). However, samples from the PRRSV-2 group taken 12 days before challenge and day 0 (the day of challenge) and those from the H3N2 group on day 0 did not follow this trend at level of phyla. At the family level, *Weeksellaceae* were, the most abundant in samples taken from the PRRSV-2 group, 12 days before viral challenge (at 18.7%).

### 2.3. The Nasal Microbiota Community Changes after Viral Infection

Similarly to the bacterial communities of the nasal swabs taken from the uninfected pigs, the profiles of the nasal swabs taken from three groups of virally challenged pigs fluctuated over the length of the experiment (Figure 2a,b). On average, the most abundant bacterial group after the challenge in the three viral challenge groups was, at the phylum level, Proteobacteria (ranging from 47.0–63%), Bacteroidetes (ranging from 11.1–18.8%), and Firmicutes (ranging from 8.7–20.2%) (Supplementary Table S1). At family level, *Moraxellaceae* (ranging from 22.7–55.1%) were the most abundant (Supplementary Table S2).

**Figure 3:**
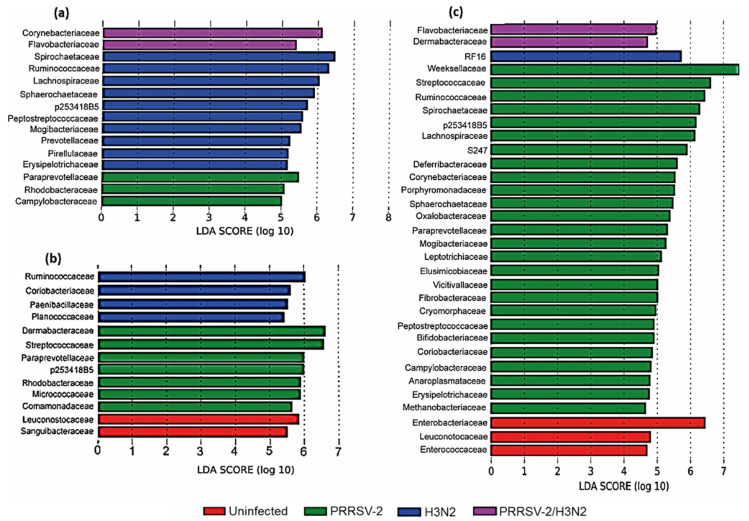
Bacterial families identified by LEfSe differing significantly in relative abundance between the four groups of pigs at different time points after viral infection. The LDA scores plots indicate the strength of the association of the bacterial family with one of the groups when comparing them at day 0 (**a**) day 3; (**b**) and day 5; (**c**) post infection.

Linear discriminant analysis Effect Size (LEfSe) was used to identify bacterial families that differed in relative abundance between the four groups of pigs in the nasal swab samples taken 0, 3 and 5 dpi (Figure 3). Immediately before infection (0 dpi), three families were shown to be increased in abundance in the PRRSV-2 group, ten families in the H3N2 group, and two families in the PRRSV-2/H3N2 group. The family *Paraprevotellaceae* was mostly strongly associated with the PRRSV-2 group, *Spirochaetaceae* with the H3N2 group and *Corynebacteriaceae* with the PRRSV-2/H3N2 group. Three days after infection, two families were demonstrated to be increased in the uninfected group, seven families in the PRRSV-2 group and four families in the H3N2 group. The family *Leuconostocaceae* was most strongly associated with the uninfected group, *Dermabacteraceae* with the PRRSV-2 group and *Ruminococcaceae* with the H3N2 group. Five days after the infection three families were increased in abundance in the uninfected group, 26 families in the PRRSV-2 group, one family in the H3N2 group and two families in the PRRSV-2/H3N2 group. *Enterobacteriaceae* were most strongly associated with the uninfected group, *Weeksellaceae* with the PRRSV-2 group, RF16 with the H3N2 group, and *Flavobacteriaceae* with the PRRSV-2/H3N2 group.

**Figure 4:**
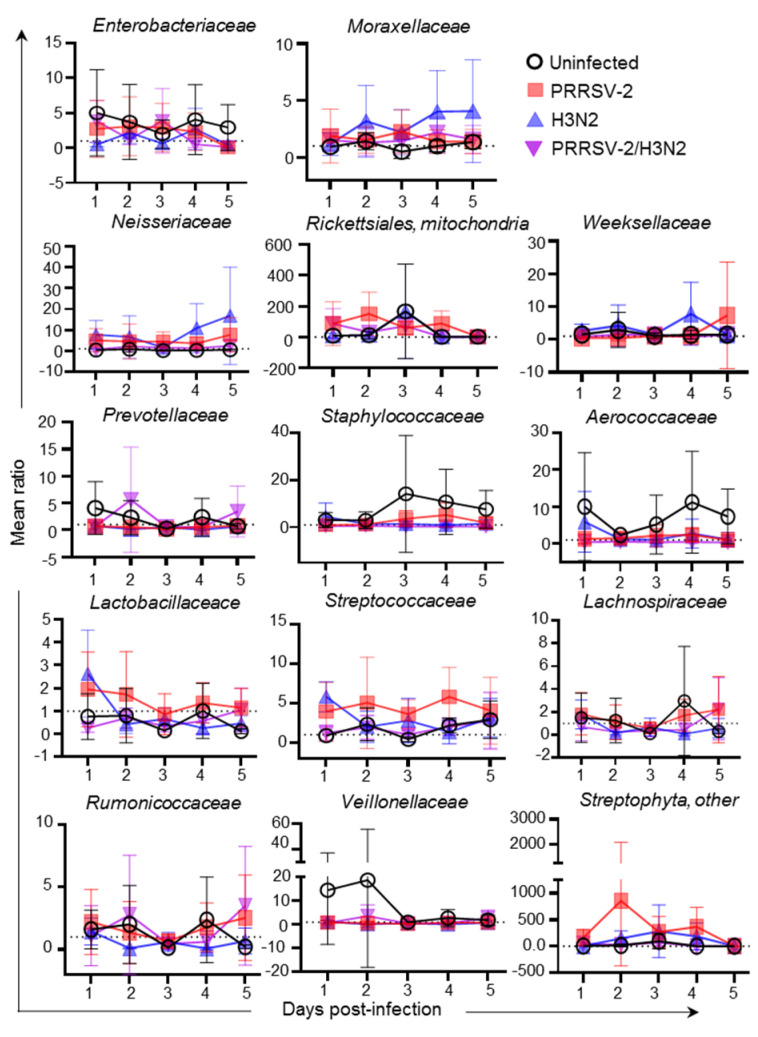
Variation of nasal microbiota in single and co-infected group. Ratio of main family after challenge at 1 to 5 dpi in comparison to the baseline (average abundance at day 0) was calculated for each animal. Each symbol represents the mean ratio of a group (*n* = 4–6 per group).

Next, we sought to determine whether the abundance of bacterial species was affected following single and co-infection. As variations in bacterial family abundance were observed between groups, the ratios of the main families were calculated after normalization to the pre-infection baseline (0 dpi) for each pig in each group (Figure 4). In the uninfected group, fluctuations of several bacterial families were observed over time. While *Enterobacteriaceae,* *Staphylococcaceae,* *Rickettsiales, mitochondria* and *Aerococcaceae* increased, *Neisseriaceae and Lactobacillaceace* decreased. In PRRSV-2 infected pigs, three bacteria families were reduced (*Rumonicoccaceae, Prevotellaceae and Veillonellaceae*) and six were increased (*Neisseriaceae*, *Rickettsiales, mitochondria,* *Streptococcaceae, Streptophyta,* *Lactobacillaceae* and *Moraxellaceae)* in comparison to the uninfected group. Of note in PRRSV-2 infected animals, *Staphylococcaceae* increased from 3 dpi, but the ratio was not as high as in the uninfected group. The *Aerococcaceae* and *Staphylococcaceae* remained stable in PRRSV-2 whereas was increased in the uninfected group. In H3N2 infected animals, only three bacterial families were increased (*Neisseriaceae, Streptophyta* and *Moraxellaceae*) and three decreased (*Rumonicoccaceae*, *Veillonellaceae* *and Prevotellaceae*) compared to the uninfected group. In the PRRSV-2/H3N2 co-infected group, two bacterial family increased (*Neisseriaceae* and *Moraxellaceae))*, two bacteria family decreased (*Staphylococcaceae* and *Aerococcaceae*) and one remained stable (*Veillonellaceae*) compared to the uninfected group.

**Figure 5:**
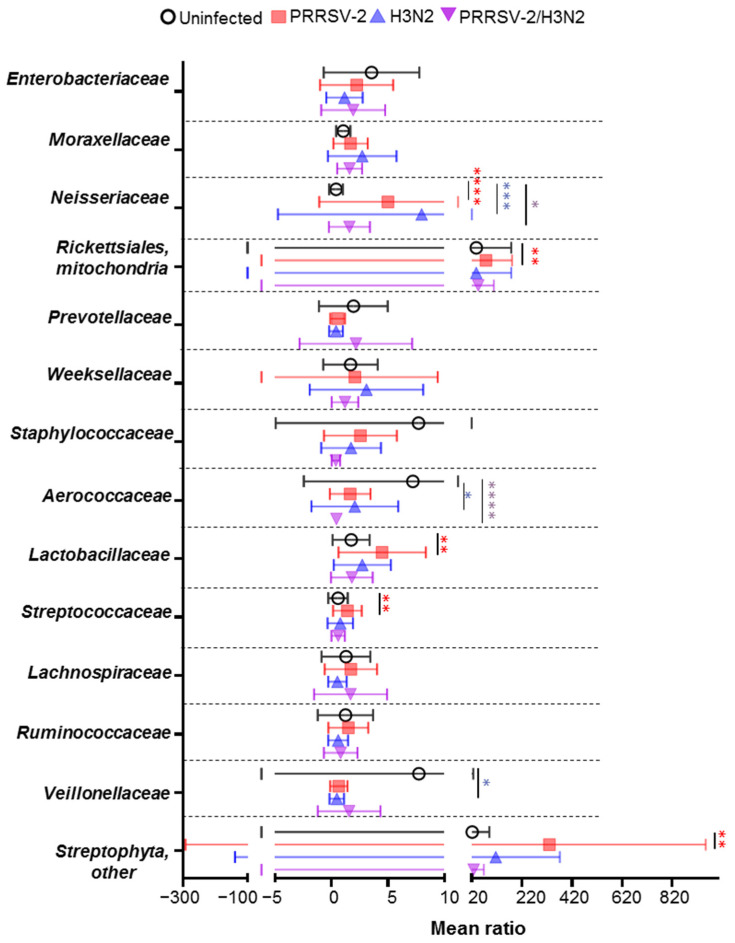
Variation of nasal microbiota in singly and co-infected groups. Average ratio of main families over five days after challenge compared to baseline (average of abundance at day 0). Each symbol represents the mean ratio of a group and bars indicate ± SD. Comparisons were made using Kruskal-Wallis test and asterisks indicate significant differences (* *p* < 0.05, ** *p* < 0.01, *** *p* < 0.001 and **** *p* < 0.0001).

To identify which bacterial families were significantly altered after infection, the average of the ratio from 1 to 5 dpi per group of pigs was calculated (Figure 5). Overall, PRRSV-2 infection significantly increased the abundance of five bacterial families in comparison to the uninfected animals (*p* < 0.05): *Neisseriaceae*, *Rickettsiales, mitochondria*, *Streptophyta, Lactobacillaceae* and *Streptococcaceae.* In H3N2 infected animals, while *Neisseriaceae* increased (*p* < 0.05), *Aerococcaceae and* *Veillonellaceae* decreased significantly (*p* < 0.05). After PRRSV-2/H3N2 co-infection, *Neisseriaceae* was also increased significantly (*p* < 0.05) and *Aerococcaceae* was significantly decreased compared to the uninfected group (*p* < 0.0001).

Altogether, these data indicate that the single infection with PRRSV-2 or H3N2 altered the distribution and abundance of bacterial species in comparison to uninfected animals. *Neisseriaceae* were increased after single and co-infection with PRRSV-2 and H3N2. The PRRSV-2/H3N2 co-infection affected the abundance of different bacterial families compared to the singly infected groups.

### 2.4. Correlation Analysis of H3N2 Titre and Relative Abundance of Bacterial Species in Nasal Swabs

As H3N2 was present in the nasal swabs (Supplementary Table S3) and a significant difference in viral load was measured at 5 dpi between singly and co-infected groups [22], we assessed the quantitative association between the H3N2 viral load and abundance of the main bacterial families (≥1% in average across the study) after infection using the Spearman’s correlation test (Table 1). For each animal in the H3N2 and PRRSV-2/H3N2 groups, the integrated values of the viral load (viral titer area under the curve (AUC)) and of the main bacterial families (percentage of bacteria family AUC)) over the 5 dpi were calculated. A total of 14 bacterial families were analyzed and the results indicated a significant positive correlation between H3N2 AUC and *Neisseriaceae* AUC (r = 0.61, *p* < 0.05) (Table 1).

## 3. Discussion

Here we examined the early changes in the porcine nasal microbiota before and after single infection with PRRSV-2 or H3N2 or co-infection with PRRSV-2 and H3N2 in pigs, and whether these alterations could be associated with disease outcome by scoring clinical signs and lung pathology, and by measurement of viral load [22].

A high level of bacterial diversity and richness has been associated with healthy pig cohorts and the removal of antibiotic use [12,24,25]. The bacterial richness and microbiota composition in the nasal cavity fluctuated over the time without any treatment. This was not surprising as inter-individual differences in the nasal microbiota from commercial reared pigs is expected and can vary from day-to-day [26,27]. The microbiota composition and diversity are dependent on both intrinsic (e.g., age) and extrinsic factors (e.g., environmental factors and sampling) [10,28], despite similar controlled housing and food diet during the study. Therefore, we analyzed changes compared to day 0. Overall, the variation of bacterial richness was similar between groups, although a slight reduction was observed after single PRRSV-2 or H3N2 infections, especially in the H3N2 group. In contrast after PRRSV-2/H3N2 co-infection, nasal bacterial richness remained stable and was comparable to the control uninfected pigs. The ability to alter the microbiota richness might depend on the virulence of the viral strains used as we have shown that alone or in combination, the PRRSV-2 and H3N2 field-isolated strains used here induced a mild disease and co-infection reduced viral loads [22].

The bacterial communities analyzed from the nasal swabs of uninfected controls fluctuated in species richness and composition with Proteobacteria and *Moraxellaceae* identified as the dominant bacterial family. This is in line with a previous study monitoring bacteria in the nasal cavity of young pigs, which also identified Proteobacteria and *Moraxellaceae* as the dominant bacterial families [10]. The greatest changes in abundance of bacterial families were observed at 5 dpi. Twenty six bacterial families were increased in the PRRSV-2 group at 5 dpi. *Ruminococcaceae* were increased in the H3N2 animals at 0 and 3 dpi and increased in the PRRSV-2 group at 5 dpi. The increase in abundance of *Ruminococcaceae* has previously been reported to be associated with the treatment of *Streptococcus suis* with fatty acids and a natural anti-inflammatory component [12]. Therefore, the increase in abundance of *Ruminococceae* may be linked with the anti-inflammatory response that occurs during bacterial or viral infections. It is established that swIAV infection induces inflammation in the respiratory tract [29], which we confirmed in lungs of H3N2 infected pigs by measuring of pro-inflammatory cytokine gene expression [22]. Here, H3N2 infection resulted in a significant decrease in *Ruminococceae. Streptococcaceae* were increased in the PRRSV-2 group at 3 and 5 dpi. This family contains a number of pathogenic species which can cause disease in pigs, with *Streptococcus* bacteria previously associated with Glässer’s disease [25] or PRDC [7].

Innate and adaptive immune responses at the epithelial barrier are involved in the fine control of commensal bacterial colonization. Despite the complexity of the crosstalk between both entities, there is evidence that the inflammatory environment can affect commensal bacterial expansion [30]. SwIAV and PRRSV have different cell tropism for epithelial cells and macrophages, respectively [31] and possess different abilities to modulate the host immune response. PRRSV-2 infection allowed the expansion of 5/14 families. After H3N2 infection, only 1/14 expanded and 2/14 families were reduced, suggesting that some bacterial species take advantage of the inflammatory environment. Viral infection, alone or in combination, induced variation of different bacterial families. Remarkably, *Neisseriaceae* significantly expanded in both PRRSV-2 and H3N2 single and co-infected groups. Their abundance was correlated with H3N2 viral load in the nasal cavity which might suggest that *Neisseria* spp. may be considered an opportunistic pathogen during infection with viral respiratory disease and are often associated with pneumonia [32]. Furthermore, we have shown that co-infection with PRRSV and IAV affects disease outcome and reducing viral loads [22]. Although the molecular mechanisms for virus interference are poorly defined, it has recently been shown that PRRSV interferes with IAV replication in vitro, by inhibiting the AMP-activated protein kinase (AMPK)-mediated autophagy signaling pathway [33], which has been demonstrated to promote IAV replication *in vitro* [34]. Further investigation of the interplay between these viruses and their effect on the nasal microbiota is required to elucidate the full impact of co-infection.

Pigs are infected by the same subtype of IAV as humans and have similar sialic acid receptor distribution in their respiratory tract and lung structure [35]. Understanding IAV infection and the role of the microbiota in this large natural animal model has enormous potential for unraveling the causal links between changes in the microbiota and immunity to infection or immunization. Recently, a study showed the association between the presence of genus *Prevotella* and *Muribaculaceae* families in the feces and a stronger antibody response to immunization against IAV in pigs [36].

To the best of our knowledge this is the first study to characterize the nasal microbiota of pigs following PRRSV-2 and H3N2 infection and co-infection. Whilst further studies should be undertaken to address causality between nasal microbiota and viral replication and disease outcomes, collectively, our data suggest that single and co-infection with PRRSV-2 and H3N2 differentially influence the porcine nasal microbiota.

## 4. Materials and Methods

### 4.1. Challenge and Nasal Swab Samples

Nasal swabs were collected from a previous study performed in a high containment facility at The Pirbright Institute [22]. The study was approved by The Pirbright Institute AWERB and conducted in accordance with the UK Animals (Scientific Procedures) Act 1986 (Project Licence P6F09D691). The pigs were sourced from a high health commercial pig farm in line with the principles outlined in the FELASA recommendations (Federation of European Laboratory Animal Science Associations recommendations of best practices for the health management of ruminants and pigs used for scientific and educational purposes-2020). Animals were pre-screened to ensure an absence of exposure to IAV and PRRSV prior to their arrival by hemagglutination inhibition test against standard IAV antigens, and antibody ELISA and RT-PCR tests against PRRSV [22]. Briefly, 24 Large White-Landrace-Hampshire cross, female pigs were randomly assigned to 4 groups of 6 pigs housed in separate rooms. Pigs were experimentally infected with contemporary field-isolated strain swIAV H3N2 CM5 and/or PRRSV-2 16CB02, both viruses isolated from pig farms in Thailand. Animals were inoculated intranasally with 4 mL of virus diluted in Dulbecco’s modified MEM (DMEM, Merck, Feltham, UK) containing 5 × 10^6^ pfu of H3N2, 10^5^ TCID_50_ PRRSV-2 or concurrently with 5 × 10^6^ pfu H3N2 and 10^5^ TCID_50_ PRRSV-2 using a MAD 300 device (Wolfe Tory Medical, Salt Lake City, UT, USA). A pilot study was performed using these inoculation doses that confirmed infection in all pigs, with induction of lung lesions without causing severe disease. A control group (uninfected) was included. Nasal swabs were collected with cotton swabs (one per nostril, Scientific Laboratory Supplies, Nottingham, UK) at −12, 0, 1, 2, 3, 4 and 5 days post-infection (dpi). On day 0 (the day of infection) the nasal swabs were collected before viral challenge. Following infection and co-infection with H3N2, a typical viral shedding pattern was observed with virus detected in nasal swabs from 1–5 dpi (Supplementary Table S3) and in the bronchoalveolar lavage fluid (BALF) (Supplementary Table S4). As expected, PRRSV-2 shedding was detected by nasal swabs later starting from 3 dpi in 4 pigs in the single infected group and only in 1 pig in the co-infected group (Supplementary Table S3). Pigs were confirmed to be infected with PRRSV-2 by assessing viral RNA loads in the BALF. All animals in both single infected group and 5/6 animals in the co-infected group showed high levels of PRRSV-2 RNA on 5 dpi (Supplementary Table S4). Nasal swabs were immediately placed into a sterile 15 mL collection tube and 1 mL of TRIzol™ Reagent (Thermo Fisher Scientific, Loughborough, UK) was added. Samples were vortexed for 30 s and centrifuged at 2300× *g* for 5 min. Supernatants were harvested and stored at −80 °C until required.

### 4.2. DNA Extraction by TRIzol and Column Purification

Aqueous, interphase and organic phase was separated by adding 200 μL of chloroform (Merck) into TRIzol samples and centrifugation for 15 min, 12,000× *g* at 4 °C. After withdrawal of aqueous phase, 600 μL of 100% ethanol was added into the tube containing interphase and organic phases. Mixtures were vortexed for 15 s and DNA was subsequently purified using QIAamp Fast DNA Stool Mini Kit (QIAGEN) according to the manufacturer’s instruction. To transfer samples from a high biocontainment laboratory to lower biocontainment for downstream microbiota analysis, chemical and heat inactivations were performed according to standard operating procedures at The Pirbright Institute. Sodium acetate (2 M pH ≤ 5.6) was added into DNA samples at a 1:10 dilution followed by 2.5× volume of absolute ethanol. DNA was further purified using Genomic DNA Clean & Concentrator (Zymo Research). Heat inactivation at 56 °C for 2 h was performed before sample transfer and shipment to the Animal and Plant Health Agency (APHA, Weybridge, UK) for 16S rRNA gene sequencing.

### 4.3. 16S rRNA Gene Amplicon Sequencing

Aliquots of extracted DNA were amplified with universal primers for the V4 and V5 regions of the 16S rRNA gene. The primers U515F (5′-GTGYCAGCMGCCGCGGTA) and U927R (5′-CCCGYCAATTCMTTTRAGT) [37] were designed to permit amplification of both bacterial and archaeal ribosomal RNA gene regions, whilst providing the best possible taxonomic resolution based on published information [38,39]. Forward and reverse fusion primers consisted of the Illumina overhang forward (5′-TCGTCGGCAGCGTCAGATGTGTATAAGAGACAG) and reverse adapter (5′-GTCTCGTGGGCTCGGAGATGTGTAATAAGAGACAG) respectively. Amplification was performed with FastStart HiFi Polymerase (Roche Diagnostics Ltd.) using the following cycling conditions: 95 °C for 3 min; 25 cycles of 95 °C for 30 s, 55 °C for 35 s, 72 °C for 1 min; followed by 72 °C for 8 min. Amplicons were purified using 0.8 × volumes of Ampure XP magnetic beads (Beckman Coulter). Each sample was then tagged with a unique pair of indices and the sequencing primer, using Nextera XT v2 Index kits, and 2× KAPA HiFi HotStart ReadyMix using the following cycling conditions: 95 °C for 3 min; 12 cycles of 95 °C for 30 s, 55 °C for 30 s, 72 °C for 30 s; followed by 72 °C for 5 min. Index-tagged amplicons were purified using 0.8 volumes of Ampure XP magnetic beads (Beckman Coulter). The concentration of each sample was measured using the fluorescence-based Quantifluor assay (Promega). Concentrations were normalized before pooling all samples, each of which would be subsequently identified by its unique index combination. Sequencing was performed on an Illumina MiSeq with 2 × 300 base reads according to the manufacturer’s instructions (Illumina, Cambridge, UK).

### 4.4. Microbiota Analysis

Sequence files were uploaded onto a remote Linux server provided by the University of Surrey and quantitative insights into microbial ecology 2 (QIIME2) was used for all processing and analyses carried out (qiime2-2020.2) [40]. Files were imported and converted into a QIIME2 file (qiime tools import). Quality control program DADA2 [41] was used to trim reads at positions 50 and 280 from the forward reads and at positions 50 and 260 from the reverse reads to remove low quality reads (qiime dada2 denoise-paired). Alignment was performed on the sequences (qiime alignment mafft) and this alignment was masked to remove positions that were highly variable (qiime alignment mask). FasTtree was used to generate a phylogenetic tree from this masked alignment (qiime phylogeny fastree) and midpoint rooting was applied (qiime phylogeny midpoint-root). Core metrics were generated at a sampling depth of 6000 reads. Alpha rarefaction boxplots using the observed_OTUs (by feature) measure were generated and significant differences in alpha rarefaction between groups were assessed (qiime diversity alpha-group-significance). The reference database greengenes [42] was utilized and trained on the sequences generated from the study (qiime feature-classifier classify-sklearn). Taxonomic composition of all samples and samples by groups were generated (qiime taxa barplot). To identify bacterial groups that differed between groups of samples the BIOM table was downloaded as text and analyzed using linear discriminate analysis effect size (LEfSe) [43]. The variation of bacterial richness per animal was calculated as ratio of total number of OTUs between a baseline (number of OTUs at 0 dpi per group) and 1, 2, 3, 4 and 5 dpi. Similar approach was used to measure the variation of bacterial family abundance using percentage of main families excluding unassigned bacteria (percentage ≥1% in at least 50% of sampling within a group).

### 4.5. Statistical Analysis

Data were analyzed with GraphPad Prism 8.0.1 software. As distribution did not follow a normal distribution (Anderson-Darling test) the non-parametric Kruskal-Wallis test followed by Dunn’s correction was applied to compare data between groups. Spearman’s test was used for correlation analysis. For each pig, the integrated H3N2 titre (pfu/mL) in the nasal swabs which corresponding to the area under the curve (AUC), and percentage of the main bacteria family AUC from day 0 to day 5 post-infection were calculated. Data were subject to statistical correlations were analysed using Spearman’s rank test.

## Figures and Tables

**Table 1:** Correlation of H3N2 titer and nasal microbiota in nasal swabs.

Bacterial Family	Spearman r Correlation
*Enterobacteriaceae*	−0.07
*Moraxellaceae*	−0.23
*Neisseriaceae*	0.61 *
*Rickettsiales, mitochondria*	−0.17
*Prevotellaceae*	0.20
*Weeksellaceae*	0.38
*Staphylococcaceae*	0.41
*Aerococcaceae*	0.56
*Lactobacillaceae*	0.40
*Streptococcaceae*	0.27
*Lachnospiraceae*	−0.42
*Ruminococcaceae*	0.27
*Veillonellaceae*	−0.39
*Streptophyta, other*	−0.28

Correlation coefficient r is indicated and significant difference are indicated with asterisks (* *p* < 0.05).

## Data Availability

The raw sequences are available through accession number PRJNA758406 on the NCBI Sequence Read Archive (SRA).

## Supplementary Materials

The following are available online at www.mdpi.com/article/10.3390/pathogens10101225/s1, Table S1: Percentage of bacteria at the phylum level in the nasal swabs are shown in the excel sheet. Table S2: Percentage of bacteria at the family level in the nasal swabs are shown in the excel sheet. Table S3: Virus load in the nasal swabs. Table S4: Virus load in the bronchoalveolar lavage fluid.
