# Supplementary material for: Changes in the Nasal Microbiota of Pigs Following Single or Co-Infection with Porcine Reproductive and Respiratory Syndrome and Swine Influenza A Viruses
Source: Pathogens. 2021 Sep 22;10(10):1225. doi: 10.3390/pathogens10101225 (PMC8540314; doi:10.3390/pathogens10101225)
Supplement: Supplementary file 1 [file pathogens-10-01225-s001.zip › Supplementary materials/Supplementary Table S3.pdf]

Table S3. Virus load in nasal swabs.

	H3N2 titer (pfu/mL)						PRRSV-2 RNA (genome copies/mL)					
	0 DPC	1 DPC	2 DPC	3 DPC	4 DPC	5 DPC	0 DPC	1 DPC	2 DPC	3 DPC	4 DPC	5 DPC
Naïve	-	-	-	-	-	-	-	-	-	-	-	-
	-	-	-	-	-	-	-	-	-	-	-	-
	-	-	-	-	-	-	-	-	-	-	-	-
	-	-	-	-	-	-	-	-	-	-	-	-
	-	-	-	-	-	-	-	-	-	-	-	-
	-	-	-	-	-	-	-	-	-	-	-	-
PRRSV-2	-	-	-	-	-	-	-	-	-	8.75E+01	1.91E+04	3.10E+04
	-	-	-	-	-	-	-	-	-	-	-	-
	-	-	-	-	-	-	-	-	-	-	2.39E+02	2.19E+02
	-	-	-	-	-	-	-	-	-	-	-	7.17E+02
	-	-	-	-	-	-	-	-	-	1.40E+03	9.32E+02	5.24E+03
	-	-	-	-	-	-	-	-	-	-	-	-
H3N2	-	5.50E+02	2.00E+04	1.75E+03	4.88E+03	8.75E+02	-	-	-	-	-	-
	-	2.50E+01	5.88E+04	8.75E+03	7.50E+03	5.25E+03	-	-	-	-	-	-
	-	7.50E+01	2.75E+03	1.10E+03	8.50E+03	2.25E+03	-	-	-	-	-	-
	-	4.50E+02	1.75E+04	9.25E+03	5.13E+04	4.25E+04	-	-	-	-	-	-
	-	5.00E+01	4.75E+04	3.25E+04	5.38E+04	2.00E+04	-	-	-	-	-	-
	-	1.50E+02	2.50E+04	4.75E+04	2.79E+04	7.00E+04	-	-	-	-	-	-
PRRSV-2 + H3N2	-	1.00E+02	3.50E+03	9.25E+03	3.25E+03	2.75E+02	-	-	-	-	-	-
	-	2.75E+04	8.00E+04	1.50E+04	1.15E+04	4.75E+02	-	-	-	2.12E+01	8.27E+03	3.63E+03
	-	2.50E+02	1.00E+04	9.13E+02	8.25E+03	2.00E+02	-	-	-	-	-	-
	-	4.75E+02	1.11E+04	7.63E+02	1.00E+04	3.75E+02	-	-	-	-	-	-
	-	7.25E+02	3.75E+04	5.50E+03	2.00E+04	1.06E+03	-	-	-	-	-	-
	-	2.75E+04	9.50E+03	3.25E+03	4.50E+04	3.75E+02	-	-	-	-	-	-

DPC: day post challenge; - not detected
