# Supplementary material for: Changes in the Nasal Microbiota of Pigs Following Single or Co-Infection with Porcine Reproductive and Respiratory Syndrome and Swine Influenza A Viruses
Source: Pathogens. 2021 Sep 22;10(10):1225. doi: 10.3390/pathogens10101225 (PMC8540314; doi:10.3390/pathogens10101225)
Supplement: Supplementary file 1 [file pathogens-10-01225-s001.zip › Supplementary materials/Supplementary Table S4.pdf]

Table S4. Virus load in bronchoalveolar lavage fluid.

	H3N2 titer (pfu/mL)	PRRSV-2 RNA (genome copies/mL)
Naïve	-	-
	-	-
	-	-
	-	-
	-	-
	-	-
PRRSV-2	-	9.94E+06
	-	1.13E+05
	-	3.51E+06
	-	1.42E+06
	-	2.75E+03
	-	3.40E+06
H3N2	-	-
	2.50E+00	-
	-	-
	5.00E+01	-
	8.00E+01	-
	1.49E+02	-
PRRSV-2 + H3N2	-	8.88E+05
	5.00E+00	3.72E+04
	-	1.35E+04
	5.00E+00	6.01E+04
	-	-
	-	3.58E+04

- not detected
